# Favorable 90-Day Mortality in Obese Caucasian Patients with Septic Shock According to the Sepsis-3 Definition

**DOI:** 10.3390/jcm9010046

**Published:** 2019-12-24

**Authors:** Caspar Mewes, Carolin Böhnke, Tessa Alexander, Benedikt Büttner, José Hinz, Aron-Frederik Popov, Michael Ghadimi, Tim Beißbarth, Dirk Raddatz, Konrad Meissner, Michael Quintel, Ingo Bergmann, Ashham Mansur

**Affiliations:** 1 Department of Anesthesiology, University Medical Center, Georg August University, D-37075 Goettingen, Germany; caspar.mewes@med.uni-goettingen.de (C.M.); boehnke.carolin@web.de (C.B.); tessa.alexander@med.uni-goettingen.de (T.A.); benedikt.buettner@med.uni-goettingen.de (B.B.); konrad.meissner@med.uni-goettingen.de (K.M.); mquintel@med.uni-goettingen.de (M.Q.); ingo.bergmann@med.uni-goettingen.de (I.B.); 2 Department of Anesthesiology and Intensive Care Medicine, Klinikum Region Hannover, D-30459 Hannover, Germany; jose.hinz@krh.eu; 3 Department of Thoracic and Cardiovascular Surgery, University Medical Center, Eberhard Karls University, D-72076 Tuebingen, Germany; aronf.popov@gmail.com; 4 Department of General and Visceral Surgery, University Medical Center, Georg August University, D-37075 Goettingen, Germany; mghadim@uni-goettingen.de; 5 Institute of Medical Bioinformatics, University Medical Center, Georg August University, D-37077 Goettingen, Germany; tim.beissbarth@ams.med.uni-goettingen.de; 6 Department of Gastroenterology and Gastrointestinal Oncology, University Medical Center, Georg August University, D-37075 Goettingen, Germany; draddat@gwdg.de

**Keywords:** obesity, septic shock, Sepsis-3 definition, 90-day mortality, Survival predictors, risk stratification

## Abstract

Septic shock is a frequent life-threatening condition and a leading cause of mortality in intensive care units (ICUs). Previous investigations have reported a potentially protective effect of obesity in septic shock patients. However, prior results have been inconsistent, focused on short-term in-hospital mortality and inadequately adjusted for confounders, and they have rarely applied the currently valid Sepsis-3 definition criteria for septic shock. This investigation examined the effect of obesity on 90-day mortality in patients with septic shock selected from a prospectively enrolled cohort of septic patients. A total of 352 patients who met the Sepsis-3 criteria for septic shock were enrolled in this study. Body-mass index (BMI) was used to divide the cohort into 24% obese (BMI ≥ 30 kg/m^2^) and 76% non-obese (BMI < 30 kg/m^2^) patients. Kaplan-Meier survival analysis revealed a significantly lower 90-day mortality (31% vs. 43%; *p* = 0.0436) in obese patients compared to non-obese patients. Additional analyses of baseline characteristics, disease severity, and microbiological findings outlined further statistically significant differences among the groups. Multivariate Cox regression analysis estimated a significant protective effect of obesity on 90-day mortality after adjustment for confounders. An understanding of the underlying physiologic mechanisms may improve therapeutic strategies and patient prognosis.

## 1. Introduction

A worldwide increasing incidence of sepsis and septic shock has had a noticeable and challenging effect on the everyday routine in intensive care units (ICUs) [1]. Despite strong efforts in early diagnosis and adequate treatment, the mortality of patients in septic shock still remains in excess of 40% [2,3,4]. We argue that a more adequate categorization of obese versus non-obese patients in septic shock would lead to an improvement of intensive care support.

According to the revised definition in 2016, sepsis is defined as an inadequate host immune response to infection, and the pathology of septic shock can be considered a severe subclass of sepsis [1]. Patients in septic shock present a “clinical construct of sepsis with persisting hypotension requiring vasopressors to maintain a mean arterial pressure (MAP) ≥ 65 mmHg and having a serum lactate level > 2 mmol/L despite adequate volume resuscitation” [1]. Overwhelming inflammation leads to cellular damage and dysfunction, such as thrombocytopenia and hyperlactatemia, and results in cardiovascular compromise [5,6]. Pulmonary hypertension, arterial hypotension and vital organs suffering from hypoperfusion are the consequence [7]. In many cases, these symptoms result in multiple organ failure or death.

The clinical phenotype and outcome of septic shock are multifactorially affected by exogenous and endogenous factors. Exogenous factors such as diagnostic procedures or antibiotic treatment strategies are still up for discussion and are often inconsistent [8,9]. Likewise, endogenous patient factors, such as the individual immune status, host genetic factors, microbiological findings and existing comorbidities, have gained attention in recent years [10,11,12,13,14,15]. In particular, the effect of obesity, one of the most common comorbidities in critically ill patients, on sepsis and septic shock has been examined in various studies [16,17]. Defined by the World Health Organization (WHO) as a BMI ≥ 30 kg/m^2^, obesity is associated with a number of other concomitant comorbidities, such as hypertension, cardiovascular diseases, diabetes mellitus type 2, non-alcoholic fatty liver disease, sleep apnea, and cancer [18,19].

Despite predominantly negative associations, previous studies have also suggested obesity as a possible protective factor in sepsis and septic shock [20,21]. Biologically active and hormone secreting adipose tissue leads to alterations at both the cellular and the systemic level [22]. A greater metabolic reserve, increased activation of the renin-angiotensin-aldosterone system (RAAS), and an altered secretion of immunomodulatory mediators are discussed as possible rationales [23,24]. However, results from previous studies regarding the effect of obesity on sepsis and septic shock have been inconsistent; these studies were mostly conducted in the context of retrospective study designs and limited sample sizes and exhibited inadequate adjustments for confounders. Furthermore, relevant studies rarely applied the actual Sepsis-3 definition for septic shock [25,26,27].

The present study, therefore, investigated a cohort of Caucasian patients with septic shock according to the current definitions and guidelines to reveal the effect of obesity on mortality and the course of disease. The primary outcome was 90-day mortality risk, whereas 28-day mortality, analyses of detailed clinical parameters, disease severity, microbiological findings, and an adequate adjustment for confounders served as secondary endpoints. Based on previous investigations, it was hypothesized that obesity could lead to better outcomes, more precisely, a favorable 90-day mortality in patients with clinically defined septic shock.

## 2. Material and Methods

### 2.1. Setting

The present study was performed at the University Medical Center Goettingen, Germany. All investigations were approved under the ethical project identification code 1/15/12 by the institutional ethics committee of the University of Goettingen, Germany. The study was performed in accordance with the provisions, relevant guidelines and regulations of the Declaration of Helsinki. Written informed consent was obtained from all patients or their legal representatives.

### 2.2. Patients

A total of 352 patients who met the International Consensus Sepsis-3 definition criteria for septic shock were selected from a prospectively enrolled cohort of 686 patients with sepsis. For the purpose of the present study, the remaining 334 patients with sepsis but not in septic shock were withdrawn from the study. All patients were classified as either non-obese (BMI < 30 kg/m^2^) or obese (BMI ≥ 30 kg/m^2^) according to the WHO definition of obesity. Patient recruitment took place at three surgical ICUs of the University Medical Center Goettingen. All adult Caucasian patients admitted to the three ICUs were screened daily for septic shock according to the current guidelines and definitions. Eligible patients were added to the study database and followed for 28 days with daily data acquisitions. Mortality within 90 days was recorded by either individual telephone follow-up or official written request at the local registry. No patient was lost to follow-up. Non-eligible patients were excluded from the study according to the following exclusion criteria: (I) immunosuppressive therapy (e.g., azathioprine, cyclosporine, glucocorticoids) or cancer-related chemotherapy, (II) myocardial infarction within six weeks before enrolment, (III) human immunodeficiency virus (HIV) infection, (IV) congestive heart failure classified as level IV by the New York Heart Association (NYHA), (V) end-stage incurable disease with a reduced probability of surviving the following 28 days, (VI) pregnancy or breastfeeding, (VII) age below 18 years, (VIII) “Do Not Resuscitate” (DNR) or “Do Not Treat” (DNT) order, (IX) persistent vegetative stage (apallic syndrome), (X) participation in interventional studies, and (XI) familial relationship with a member of study team.

### 2.3. Data Collection

All patient data were recorded using clinical report forms (CRFs) and organized in the GENOSEP database, a database that was previously described and primarily used for genetic association studies with the clinical phenotype of sepsis [12]. Collected data included patient baseline characteristics such as gender, age in years, baseline Sequential Organ Failure Assessment (SOFA) score and Acute Physiology and Chronic Health Evaluation (APACHE II) score as well as additional variables including relevant parameters for organ-specific SOFA subscores and the following comorbidities: arterial hypertension, chronic obstructive pulmonary disease (COPD), renal dysfunction, insulin-dependent diabetes mellitus (IDDM), non-insulin-dependent diabetes mellitus (NIDDM), and history of cancer. All parameters were collected for up to 28 days unless the patient died or left the ICU at an earlier stage. The collection of disease severity parameters included organ-specific SOFA subscores and measures of organ support, such as ventilated days, days with catecholamine therapy (any dose of norepinephrine, epinephrine or dobutamine), dialyzed days (including continuous renal replacement therapy), and their proportions to observation days was reported as percentages. In addition, inflammatory values such as leukocyte count, C-reactive protein (CRP) and procalcitonin levels as well as kidney values (creatinine or urine output) and central nervous system (CNS) Glasgow Coma Score (GCS) were recorded. All clinical data and relevant microbiological findings were gathered from the electronic patient record system (IntelliSpace Critical Care and Anesthesia (ICCA), Philips Healthcare, Andover, MA, USA).

### 2.4. Statistical Analyses

All statistical analyses were performed using Statistica 13 software (version 13.0, StatSoft, Tulsa, OK, USA). Tables in this paper show continuous variables presented as the mean values ± the standard deviations or as median and interquartile ranges (IQRs) and categorical variables as absolute numbers or percentages as marked in the respective columns. *p*-values ≤ 0.05 were considered statistically significant. The significance for continuous variables was calculated using the Mann-Whitney U-test or Kruskal-Wallis test, if applicable. Categorical variables were examined using the Pearson chi-square test or the two-sided Fisher’s exact test. Furthermore, time-to-event data were compared with the log-rank test in the Kaplan-Meier survival analysis. A multivariate Cox regression analysis was applied to the observed time-to-event survival data in order to estimate proportional hazard ratios and adjust for relevant confounders. A power calculation was conducted using the Statistica 13 software package for power analysis considering a probability of a type I error (alpha) of 0.05.

### 2.5. Availability of Data and Materials

The datasets used and analyzed during the current study are available from the corresponding author upon reasonable request.

## 3. Results

### 3.1. Baseline Characteristics

A total of 352 adult Caucasian patients met the criteria for septic shock and were enrolled in the study. The cohort included 266 non-obese (76%; BMI < 30 kg/m^2^) and 86 obese patients (24%; BMI ≥ 30 kg/m^2^). Illustrated in Table 1, the average age was 65 ± 14 years, and 64% were male. In total, 141 out of 352 patients (40%) did not survive the 90-day follow-up period. The average SOFA score on the day of enrolment was significantly higher in obese patients compared to that in the non-obese patients (11.9 ± 3.7 vs. 11.0 ± 3.7; *p* = 0.023; Table 1); however, there were no significant differences found for the APACHE II scores or baseline procalcitonin levels (n = 197). The incidence rates of common comorbidities such as COPD, renal dysfunction, IDDM, and history of cancer were similar among obese and non-obese patients, except arterial hypertension, which showed a significantly higher frequency in obese patients (64% vs. 50%; *p* = 0.0206; Table 1). Furthermore, obese patients required more renal replacement therapy at baseline (22% vs. 13%; *p* = 0.0358; Table 1) as well as during the observation period (47% vs. 34%; *p* = 0.0342; Table 1) in comparison to non-obese patients. The patients’ recent surgical history did not significantly differ between the two groups.

### 3.2. Outcomes

**Figure 1:**
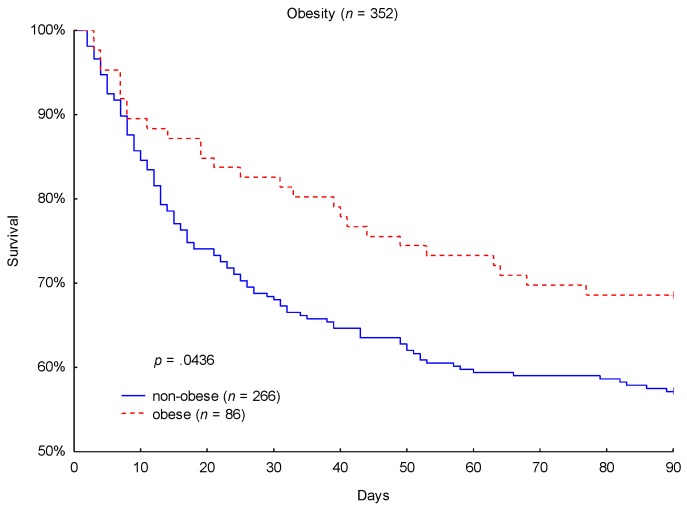
Kaplan-Meier 90-day mortality analysis with respect to obesity category.

Kaplan-Meier 90-day survival analysis was performed to obtain the primary outcome. Obese patients showed a significantly lower 90-day mortality (31% vs. 43%; *p* = 0.0436; Figure 1) in comparison to non-obese patients. While 27 out of 86 obese patients (31%) died within the considered 90-day period, 114 out of 266 non-obese patients (43%) did not survive this duration. As a secondary endpoint, a lower 28-day mortality (17% vs. 31%; *p* = 0.0159) in obese patients was also observed.

### 3.3. Disease Severity

To examine patient disease severity, the average SOFA score, organ-specific SOFA subscores and organ support, inflammatory, kidney, liver, central nervous, and coagulation parameters were compared (Table 2). Obese patients presented significantly higher average renal SOFA scores (1.4 ± 1.4 vs. 1 ± 1.3; *p* = 0.0047) over the observation period. Creatinine levels were, on average, higher (1.7 ± 1 mg/dL vs. 1.3 ± 0.9 mg/dL; *p* = 0.0002), the urine output was lower (1.2 ± 0.8 mL/kg/h vs. 1.5 ± 0.8 mL/kg/h; *p* = 0.0014), and the average number of cumulative days with renal replacement therapy was almost doubled (4.4 ± 6.9 vs. 2.4 ± 5.1; *p* = 0.0264) in obese patients compared to non-obese patients. The average inflammatory, liver, central nervous, and coagulation parameters are presented in Table 2, but no significant differences between the groups were observed. Furthermore, the length of stay (LOS) in the ICU or hospital and the average days in septic shock were similar between obese and non-obese patients.

### 3.4. Microbiological Findings

The site of infection as well as the infection type and the spectrum of pathogenic agents associated with nosocomial infections were examined. Neither the site nor the type of infection showed significant differences between the obese and non-obese patients. However, obese patients presented with significantly lower rates of *Enterococcus faecalis* infections (15% vs. 27%; *p* = 0.0286; Table 3) than non-obese patients.

### 3.5. Multivariate Cox Regression Analysis

A multivariate Cox regression analysis was conducted to exclude the possible effects of baseline characteristics or significant confounders on 28-day and 90-day mortality. Relevant baseline characteristics, such as age, gender, SOFA, and APACHE ΙΙ scores at baseline were included in the multivariate analysis as correlations to septic shock outcome were presumed based on results from previous investigations. The presence of arterial hypertension as a pre-existing medical condition, as well as the need for renal replacement therapy at baseline and during the observation period, were included as potential confounders due to the results of the patient baseline characteristics (Table 1). The multivariate model revealed obesity as a significant and independent positive predictor for 28- and 90-day survival in patients with septic shock after adjustment for confounders. Compared to non-obese patients, the adjusted hazard ratio (95% CI) of 28-day mortality was 0.462 (0.264–0.808) and of 90-day mortality was 0.57 (0.371–0.874) for obese patients (*p* = 0.0068 and *p* = 0.01, respectively; Table 4) relative to non-obese patients.

## 4. Discussion

The incidence of obesity has nearly tripled since 1975, according to the WHO, and this increase has been accompanied by rising health care costs and increasing associated comorbidities such as diabetes mellitus or arterial hypertension, which have turned this disease into a public and economic burden [28,29]. The implications of obesity for intensive care medicine, and particularly for critically ill patients with sepsis or septic shock, have been previously studied, but their interpretations were limited and inconsistent for patients sustaining septic shock according to the current Sepsis-3 definition [25,26,27].

The present study investigated the effect of obesity on 90-day mortality among Caucasian patients with clinically defined septic shock. Based on previous investigations, a better outcome and course of disease among the obese patients was hypothesized a priori.

The primary finding of this investigation was that obese patients with septic shock had a significantly better 90-day mortality rate compared to non-obese patients. In our cohort of 352 patients in septic shock, 90-day mortality was 31% for obese patients sustaining septic shock compared to 43% in non-obese patients sustaining septic shock (*p* = 0.0436). As a secondary observation, 17% of obese patients died within the first 28 days, whereas 31% of non-obese patients did not survive this period (*p* = 0.0159). To the best of our knowledge, this investigation is the first to present both a favorable short-term 28-day mortality as well as a favorable 90-day mortality in obese patients within a cohort of Caucasian patients in septic shock conforming with the 2016 Sepsis-3 definitions [1]. Furthermore, the presence of obesity remained an independent predictor for 90-day mortality (hazard ratio: 0.57; 95% CI: 0.371-0.874; *p* = 0.01) and 28-day mortality (hazard ratio: 0.462, 95% CI: 0.264–0.808; *p* = 0.0068) after adjusting for relevant baseline characteristics and significant confounders (age, gender, SOFA score at baseline, APACHE II score, diagnosed arterial hypertension, and renal replacement therapy at baseline and during observation).

Our findings are consistent with previous studies showing a protective impact of obesity on mortality in critically ill patients [20,21,30,31,32]. However, the physiologic mechanisms behind these associations remain unclear. Possible explanations include the beneficial effect of having higher metabolic reserves during a critical illness [23,33]. It may be plausible that access to adipose tissue during the highly catabolic state of septic shock is helpful to prevent severe complications or mortality. Furthermore, adipose tissue not only stores excess calories as triglycerides but also modulates the host immune response by expressing inflammatory mediators such as leptin or soluble tumor necrosis factor-receptor-2 [22,34,35]. Leptin has previously been shown to have a modulatory function on processes of the innate and adaptive immune response, such as immune cell proliferation, cytokine production, and endothelial function [36,37]. In their animal study, Siegl et al. found that relative hyperleptinemia due to obesity is protective in sepsis, presumably by improving the cellular immune response as well as by reducing the pro-inflammatory cytokine response [38].

As a further secondary endpoint, we found that obese patients showed a significantly higher incidence of arterial hypertension at baseline. The positive association between obesity and arterial hypertension has already been described in many epidemiological studies, with high correlations between BMI and arterial blood pressure values [28,29,39].

In addition, obese patients appeared to have significantly higher baseline SOFA scores as well as a higher need for renal replacement therapy both at baseline and during observation. A higher risk of sustaining chronic kidney disease and the requirement of renal replacement therapy have also been previously described in obese patients [40].

Investigations of disease severity have revealed that obese patients presented significantly higher average SOFA renal scores and creatinine levels, lower urine outputs and required renal replacement therapy for almost twice as many days as non-obese patients. Although obese patients showed a higher severity of septic shock, especially regarding kidney function, the severity did not alter the favorable outcome for 28-day and 90-day survival. These findings are consistent with previous studies in which health status at baseline and disease severity did not seem to explain the “obesity paradox” in obese critically ill patients [41].

Another finding that can be obtained from the microbiological analyses performed in this investigation was that obese patients had significantly fewer *Enterococcus faecalis* infections than non-obese patients (15% vs. 27%; *p* = 0.0286). Further research is necessary to investigate the assumption that obese patients in septic shock might be less vulnerable to secondary nosocomial infections, such as *Enterococcus faecalis*, due to a stronger immune response compared to non-obese patients. It has been suggested that an increased risk of infection is associated with reduced levels of leptin, e.g., in malnourished individuals [42].

While the relatively large cohort of patients with septic shock, the prospective data collection, and the large number of recorded clinical parameters are strengths of this investigation, there are certainly some limitations to this study. The monocentric study design and the entirely Caucasian septic shock study population are limitations of the present investigation; the results may be limited in their generalizability, especially for other ethnicities. It is of interest to assess the observed effects in cohorts of other ethnicity to allow generalization of the clinical relevance of our findings. Similarly, the studied cohort consists of critical ill surgical patients, so that some of the observations may not be representative for other ICUs (e.g., medical ICU). Thus, our findings should be further verified in cohorts from other ICUs and centers. Furthermore, a power calculation to determine a sample size with adequate power could not be conducted at the beginning of the investigation, because the effect size of obesity on the 90-day mortality in patients with septic shock according to the Sepsis-3 definition criteria was unknown. However, a performed post-hoc power analysis revealed a power of 0.86 considering the observed 90-day mortality rates of 31% and 43% among the obese and non-obese patients, respectively. Thus, our sample size was sufficiently large to adequately address the present study’s objective. The presented results are associations and do not imply causality. The physiological and molecular mechanisms behind the presented findings should be further investigated. As this study only dichotomously investigated the differences between obese and non-obese patients, a more detailed categorization of BMI (e.g., according to defined WHO adiposity grades) would be helpful to reveal potential underlying correlations in future investigations.

## 5. Conclusions

Our investigation provides important evidence for obesity as an independent prognostic variable for the survival of Caucasian patients with septic shock. In the studied cohort, the condition of obesity was independently associated with a favorable 90-day and, secondarily, 28-day outcome. We suggest using an adequate categorization of obese versus non-obese patients for risk stratification of patients in septic shock. The revealed associations need to be validated in other cohorts, and the underlying physiological mechanisms need to be revealed. The understanding of how obesity improves survival in septic shock could affect therapeutic strategies and prognosis for many patients.

## Figures and Tables

**Table 1:** Patient baseline characteristics with respect to obesity category.

Variable	All (*n* = 352)	Obese (*n* = 86)	Non-obese (*n* = 266)	*p* Value
**Demographics**				
• Age (years)	65 ± 14	63 ± 13	65 ± 15	0.1917
• Male (%)	64	63	64	0.8018
**Severity at enrollment**				
• Sequential Organ Failure Assessment (SOFA)	11.2 ± 3.7	11.9 ± 3.7	11 ± 3.7	0.023
• Acute Physiology and Chronic Health Evaluation (APACHE II)	24 ± 7	24 ± 7	24 ± 7	0.3451
• Procalcitonin [ng/dL]	2.1 (0.7–12.1) (*n* = 197)	3.25 (0.9–12.9) (*n* = 48)	1.9 (0.6–10.2) (*n* = 149)	0.3124
**Comorbidities (%)**				
• Art. hypertension	53	64	50	0.0206
• History of myocardial infarction	8	9	7	0.513
• COPD	16	21	14	0.1191
• Bronchial asthma	3	3	3	0.6776
• Renal dysfunction	11	12	11	0.7748
• Insulin-dependent diabetes mellitus (IDDM)	12	16	10	0.1856
• Non-insulin-dependent diabetes mellitus (NIDDM)	9	10	8	0.5325
• Chronic liver disease	7	9	7	0.4345
• History of cancer	16	13	18	0.2891
• History of stroke	5	5	5	0.9293
• Dementia	2	0	2	0.1038
**Recent surgical history [%]**				
• Elective surgery	29	30	28	
• Emergency surgery	21	26	20	0.3771
• No surgery	50	44	52	
**Organ support at baseline [%]**				
• Mechanical ventilation	89	92	88	0.3177
• Use of catecholamines	88	87	88	0.9253
• Renal replacement therapy	15	22	13	0.0358
**Organ support during the observation period [%]**				
• Mechanical ventilation	96	95	96	0.713
• Use of catecholamines	100	100	100	1
• Renal replacement therapy	37	47	34	0.0342

**Table 2:** Disease severity with respect to obesity category.

Parameter	All (*n* = 352)	Obese (*n* = 86)	Non-Obese (*n* = 266)	*p* Value
**SOFA scores and SOFA subscores during observation**				
• SOFA	8.7 ± 3.7	9 ± 3.9	8.7 ± 3.7	0.6074
• SOFA respiratory score	2.2 ± 0.7	2.3 ± 0.7	2.2 ± 0.7	0.3778
• SOFA cardiovascular score	2.0 ± 1	2.0 ± 1	2.1 ± 1	0.3208
• SOFA CNS score	2.3 ± 1	2.1 ± 1	2.3 ± 1	0.0743
• SOFA renal score	1.1 ± 1.3	1.4 ± 1.4	1.0 ± 1.3	0.0047
• SOFA coagulation score	0.6 ± 0.7	0.5 ± 0.7	0.6 ± 0.8	0.7871
• SOFA hepatic score	0.6 ± 0.8	0.7 ± 1	0.5 ± 0.8	0.1241
**Organ support**				
• Days with ventilation	13 ± 9	13 ± 8	13 ± 9	0.6476
• Days with catecholamines	8 ± 6	8 ± 6	8 ± 6	0.4037
• Days with dialysis	0 (0-4)	0 (0-7)	0 (0-3)	0.0264
• Days with ventilation/observation days [%]	73 ± 30	73 ± 29	73 ± 30	0.7834
• Days with catecholamines/observation days [%]	49 ± 29	49 ± 29	49 ± 29	0.9635
• Days with dialysis/observation days [%]	0 (0–25)	0 (0–39)	0 (0–21)	0.0723
**Inflammatory values**				
• Leukocytes [1000/µL]	14 ± 6	13 ± 6	14 ± 5	0.0738
• CRP [mg/L]	156 ± 80	149 ± 70	159 ± 83	0.6046
• Procalcitonin [ng/Dl]	7 ± 13	7 ± 13	7 ± 13	0.1565
**Kidney values**				
• Urine output [mL/day]	2722 ± 1536	2883 ± 1770	2670 ± 1452	0.3166
• Urine output [mL/kg/h]	1.4 ± 0.8	1.2 ± 0.8	1.5 ± 0.8	0.0014
• Creatinine [mg/dL]	1.4 ± 1	1.7 ± 1	1.3 ± 0.9	0.0002
**Liver values**				
• AST (GOT) [IU/L]	66 (39–128)	83 (44–181)	63 (38–123)	0.107
• ALT (GPT) [IU/L]	43 (22–97)	48 (25–108)	43 (22–93)	0.5006
• Bilirubin [mg/dL]	0.8 (0.5–1.6)	0.9 (0.5–1.9)	0.7 (0.5–1.5)	0.0732
**Central nervous system**				
• Glasgow Coma Score	9 ± 3	10 ± 3	9 ± 3	0.1229
**Coagulation**				
• Thrombocytes [1000/µL]	255 ± 142	248 ± 121	257 ± 148	0.9951
**Length of Stay (LOS) [days]**				
• ICU-LOS	19 (10–30)	18 (11–33)	19 (10–30)	0.6082
• Hospital-LOS	33 (20–57)	35 (21–60)	33 (20–56)	0.7073
• Days in septic shock	2 (1–4)	2 (2–4)	2 (1–4)	0.4197

**Table 3:** Microbiological findings with respect to obesity category.

	All (*n* = 352)	Obese (*n* = 86)	Non–Obese (*n* = 266)	*p* Value
**Site of infection [%]**				
• Lung	53	52	53	0.3155
• Abdomen	26	23	27	
• Bone or soft tissue	5	9	3	
• Surgical wound	2	2	2	
• Urogenital	3	1	3	
• Primary bacteraemia	8	9	7	
• Other	4	2	5	
**Type of infection [%]**				
• Bacterial (gram-positive)	79	78	80	0.7216
• Bacterial (gram-negative)	66	63	67	0.4828
• Fungal	64	71	62	0.1194
• Viral	15	15	15	0.947
**Spectrum of pathogenic agents [%]**				
• Staphylococcus aureus	21	20	21	0.7424
• Staphylococcus epidermidis	36	37	35	0.7531
• Enterococcus faecalis	24	15	27	0.0286
• Enterococcus faecium	20	21	19	0.7212
• Escherichia coli	30	26	32	0.2919
• Klebsiella pneumoniae	10	7	11	0.2525
• Pseudomonas aeruginosa	17	13	18	0.2274
• Candida albicans	35	43	32	0.0707
• Candida glabrata	16	17	16	0.7177

**Table 4:** Cox regression analysis with respect to obesity category.

	28–Day Mortality			90–Day Mortality		
Variable	Hazard Ratio	95% Cl	*p* Value	Hazard Ratio	95% Cl	*p* Value
• Age	1.025	1.008–1.043	0.0039	1.024	1.009–1.039	0.0013
• SOFA score at baseline	1.016	0.948–1.089	0.6556	1.009	0.953–1.069	0.755
• APACHE II score	1.008	0.967–1.051	0.7033	1.004	0.97–1.04	0.822
• Male gender	1.01	0.661–1.542	0.9639	0.93	0.656–1.318	0.6833
• Hypertension	0.993	0.651–1.515	0.9737	1.107	0.773–1.583	0.5803
• Dialysis at baseline	0.603	0.333–1.09	0.0937	0.611	0.37–1.007	0.0534
• Dialysis during observation	2.798	1.727–4.533	0.0001	2.769	1.841–4.165	0.0001
• Obesity (BMI ≥ 30 kg/m^2^)	0.462	0.264–0.808	0.0068	0.57	0.371–0.874	0.01
